# Nutritional value of a partially defatted and a highly defatted black soldier fly larvae (*Hermetia illucens* L.) meal for broiler chickens: apparent nutrient digestibility, apparent metabolizable energy and apparent ileal amino acid digestibility

**DOI:** 10.1186/s40104-017-0181-5

**Published:** 2017-06-01

**Authors:** Achille Schiavone, Michele De Marco, Silvia Martínez, Sihem Dabbou, Manuela Renna, Josefa Madrid, Fuensanta Hernandez, Luca Rotolo, Pierluca Costa, Francesco Gai, Laura Gasco

**Affiliations:** 1 Department of Veterinary Sciences, University of Turin, Largo Paolo Braccini 2, 10095 Grugliasco, TO Italy; 2 Department of Animal Production, University of Murcia, Campus de Espinardo, 30071 Murcia, Spain; 3 Department of Agricultural, Forest and Food Sciences, University of Turin, Largo Paolo Braccini 2, 10095 Grugliasco, TO Italy; 4 Institute of Science of Food Production, National Research Council, Largo Paolo Braccini 2, 10095 Grugliasco, TO Italy

**Keywords:** Amino acid, Apparent digestibility, Black soldier fly meal, Broiler chicken, Metabolizable energy

## Abstract

**Background:**

The study aimed to determine the apparent total tract digestibility coefficients (ATTDC) of nutrients, the apparent metabolizable energy (AME and AMEn) and the amino acid (AA) apparent ileal digestibility coefficients (AIDC) of a partially defatted (BSFp) and a highly defatted (BSFh) black soldier fly larvae meal. The experimental diets were: a basal diet and two diets prepared by substituting 250 g/kg (w/w) of the basal diet with BSFp or BSFh, respectively.

**Results:**

Significant differences were found between BSFp and BSFh meals for ATTDC of the nutrients: BSFp resulted more digestible than BSFh, except for ATTDC of CP which did not differed between meals, while a statistical trend was observed for ATTDC of DM and EE. The AME and AMEn values were significantly (*P* < 0.05) different between the two BSF meals, with higher levels for BSFp (16.25 and 14.87 MJ/kg DM, respectively). The AIDC of the AA in BSFp ranged from 0.44 to 0.92, while in BSFh they ranged from 0.45 to 0.99. No significant differences were observed for the AA digestibility (0.77 and 0.80 for BSFp and BSFh, respectively), except for glutamic acid, proline and serine that were more digestible in the BSFh meal (*P* < 0.05).

**Conclusions:**

Defatted BSF meals can be considered as an excellent source of AME and digestible AA for broilers with a better efficient nutrient digestion. These considerations suggested the effective utilization of defatted BSF larvae meal in poultry feed formulation.

## Background

World population is expected to grow by over a third, reaching over 9 billion people in 2050 having as main consequence that the world will have to produce 70% more food [1]. Consequently, livestock production (in particular that of poultry and swine) will grow exponentially reaching up to double of the current production. Therefore, the foremost gamble will be to guarantee the global capacity to provide enough animal feed (in particular protein ingredients) trying to avoid as much as possible competition with human food demand. For this purpose, insects have been already proposed as a high quality, efficient and sustainable alternative protein source for poultry [2], fish [3–5] or swine [6]. One of the most promising insects species identified for industrial production in the Western world is the black soldier fly (BSF, *Hermetia illucens* L.). This insect is normally reared on materials that are unsuitable for human nutrition (e.g., by-products from food processing, organic waste) [7, 8], reaching high growth rates with a good feed conversion [9]. Using different rearing substrates derived from food manufacturing (beet molasses, potato steam peelings, spent grains and beer yeast, bread remains, and cookie remains) and differing in lipid and fat contents, BSF showed a feed conversion rate that ranged between 1.4 and 2.6 [9]. BSF is able to convert these substrates in high quality protein, the content of which ranges from 38% to 46% of DM [9]. The amino acids (AA) composition of BSF is rich in methionine and lysine (9.05 and 22.3 g/kg DM, respectively) [9], and is reported to be similar or even superior than that of soybean [10]. However, nowadays knowledge about the suitability of the use of BSF as poultry feed ingredient is scarce and little-to-date. At this regard, acceptability by animals, feed conversion rates, animal health issues and quality of the obtainable animal-derived food products are of particular interest and not yet investigated sufficiently [11]. In general, little information on the digestibility of insects in livestock species is currently available. Published data on the apparent metabolizable energy (AME) of BSF for broilers are limited as also those on the ileal amino acid digestibility [12]. That limitation consisted on this information is often obtained from a whole insect meal without separation of the fat fraction from the protein one. As it is known, for an efficient and optimal broiler feed formulation the best situation is to manage fat and protein sources separately. For this purpose, and because insect farming is going to be a growing market, nowadays BSF manufacturers have started to produce defatted BSF meals. Defatting of insects can be obtained mechanically by cutting the frozen insect larvae and then pressing them to enable the leakage of intracellular fat [13], or chemically using petroleum ether extraction of the insect meal [14]. The defatting process results in meals with larger protein values (476 to 583 g/kg DM for defatted BSF meals) [13, 15], exceeding those usually found in soybean meals [14, 16, 17]. Henry et al. [5] suggested the importance of defatting insect meals, then using insect protein concentrate as animal feed ingredient and the lipids both for animal nutrition and the production of biodiesel [18, 19].

In order to provide new information useful to facilitate poultry feed formulation, the aim of this study was to determine the total tract digestibility coefficients (ATTDC), the AME, the nitrogen-corrected AME (AMEn) and the AA apparent ileal digestibility coefficients (AIDC) of a partially and a highly defatted BSF meal for broiler chickens.

## Methods

The study was performed at the poultry facility of the Department of Agricultural, Forest and Food Sciences of the University of Turin (Italy). The experimental protocol was designed according to the guidelines of the current European and Italian laws on the care and use of experimental animals (European Directive 86/609/EEC, put into law in Italy with D.L. 116/92) and was approved by the Ethical Committee of the Department of Veterinary Sciences of the University of Turin (Italy) (protocol number 01/28/06/2016).

### Insect meals

Two BSF meals, differing for their fat content, were used in this trial. Both insect meals were obtained from Hermetia Deutschland GmbH & Co. KG, Baruth/Mark (Germany). The two BSF meals derived from larvae which were fed with cereal by-products. At collection, the larvae weighed around 150–220 mg. The collected larvae were dried for 20 h in an oven at low temperature (60 °C) and ground to a meal. BSF meals were partially and highly defatted. The process was performed using high pressure and without solvents.

### Pre-experimental period

A total of one hundred one-day-old male broiler chickens (Ross 308) were raised in a floor pen till d 21 and fed a commercial broiler starter diet (227 g/kg of crude protein (CP); 13.4 MJ/kg of AME). All the birds were vaccinated at hatching against Newcastle disease, Marek disease, infectious bronchitis and coccidiosis. At d 21, sixty birds of uniform body weight (822.0 ± 47.7 g) were chosen and randomly distributed over fifteen cages (4 birds per cage). The cages (120 cm × 60 cm) were placed in an insulated room provided with devices to control temperature, light and humidity. Each cage had a linear feeder at the front and a nipple drinker at the back. Health status and mortality of the birds were monitored daily throughout the whole experimental period. From d 21, the birds were fed a commercial broiler diet (190 g/kg of CP; 13.6 MJ/kg AME) until the assay diets were introduced on d 26. During the pre-experimental period, feeds and water were provided *ad libitum*.

### Digestibility trial

On d 26, five replicate cages were randomly assigned to one out of three experimental diets. A basal diet, based on corn and soybean meal, was formulated (Table 1), and two experimental diets were subsequently formulated by substituting 250 g/kg (w/w) of the basal diet with BSFp or BSFh, respectively. All diets contained titanium oxide (TiO, 5 g/kg) as an indigestible marker to calculate the ileal digestibility of amino acids. The diet adaptation period lasted 6 d. Starting at the age of 32 d, the excreta were collected per cage over a period of four days in order to evaluate the total tract digestibility. Fresh feeds and water were available ad libitum during the whole experimental period. Feed intake per cage was measured throughout the experiment and the excreta were sampled daily during the test period. The total fresh excreta per cage was weighed daily, frozen at −20 °C and lyophilized. Four days excreta per cage were pooled for further analysis.

**Table 1:** Composition (g/kg as fed) of the basal diet

Ingredients	
Maize meal	582.9
Soybean meal	350.0
Soybean oil	36.0
Dicalcium phosphate	12.4
Calcium carbonate	11.2
Sodium chloride	2.0
Sodium bicarbonate	1.5
Trace mineral-vitamin premix^a^	4.0
Calculated analysis	
AME, MJ/kg	12.3
Crude protein	214
Methionine	1.0
Lysine	10.1
Threonine	7.1
Calcium	8.8
Phosphorous	5.8

*AME* apparent metabolizable energy

^a^Mineral-vitamin premix (Final B Prisma, IZA SRL), given values are supplied per kg diet: 2,500,000 IU of vitamin A; 1,000,000 IU of vitamin D_3_; 7,000 IU of vitamin E; 700 mg of vitamin K; 400 mg of vitamin B_1_; 800 mg of vitamin B_2_; 400 mg of vitamin B_6_; 4 mg of vitamin B_12_; 30 mg of biotin; 3,111 mg of Ca pantothenate acid; 100 mg of folic acid; 15,000 mg of vitamin C; 5,600 mg of vitamin B3; 10,500 mg of Zn, 10,920 mg of Fe; 9,960 mg of Mn; 3850 mg of Cu; 137 mg of I; 70 mg of Se

On d 35, all the birds were euthanized by intravenous injection of sodium pentobarbital and the content of the lower half of the ileum was collected, according to the procedures described by Ravindran et al. [20]. The ileum was defined as the portion of small intestine extending from Meckel’s diverticulum to a point 40 mm proximal to the ileo-cecal junction. The ileal content for each cage was pooled, lyophilized, ground to pass through a 0.5-mm sieve, and stored at −20 °C in airtight containers until laboratory analyses were conducted.

### Chemical analysis

Insect meals, diet samples and dried excreta were ground to pass through a 0.5-mm sieve and stored in airtight plastic containers. They were analyzed for DM (# 930.15), ash (# 924.05), CP (# 984.13) and ether extract (EE, # 920.39) according to AOAC procedures [21]. Gross energy (GE) was measured using an adiabatic calorimetric bomb (C7000, IKA, Staufen, Germany). Chitin was analyzed as D-Glucosamine [22] using a modification of method described by Madrid et al. [23] for AA. The uric acid (UA) content in the excreta samples was determined spectrophotometrically (UNICAN UV–Vis Spectrometry, Helios Gamma, United Kingdom) according to Marquardt method [24]. The CP amount of excreta was calculated as follows: CP = (total nitrogen – UA-nitrogen) × 6.25. All analyses were carried out on three replicates for each sample.

The apparent digestibility trial was performed, using the total excreta collection method, to determine ATTDC for DM, organic matter (OM), CP, EE, GE and the AME.

Ileal content samples from each cage were analyzed for DM and AA. In order to perform the AA determination, samples of the diets, ileal digesta and insect larvae meals were prepared using a 22-h hydrolysis step in 6 NHCl at 112 °C under a nitrogen atmosphere. The AA in hydrolysate was determined by HPLC (Waters Alliance System with a Waters 1525 Binary HPLC pump, Waters 2707 autosampler and Waters 2475 multi λ Fluorescence Detector, Milford, USA) after derivatization, according to the procedure described by Madrid et al. [23]. Tryptophan was not determined. Diets samples and ileal content samples were analyzed for TiO content. TiO content was measured on a UV spectrophotometer (UNICAN UV–vis Spectrometry, Helios Gamma, United Kingdom) following the method of Short et al. [25].

### Calculations

The ATTDC of the dietary nutrients, the AME and the AIDC of the AA were calculated using two different methods [20, 26].

The ATTDC of the dietary nutrients of the insect larvae meals were calculated as follows:

$$ \mathrm{ATTDC}\ {\mathrm{X}}_{\mathrm{diet}} = \left[\left(\mathrm{total}\ \mathrm{X}\ \mathrm{ingested}\hbox{--}\ \mathrm{total}\ \mathrm{X}\ \mathrm{excreted}\right)\ /\ \mathrm{total}\ \mathrm{X}\ \mathrm{ingested}\right] $$

$$ \mathrm{ATTDC}\ {\mathrm{X}}_{\mathrm{insect}\ \mathrm{larvae}\ \mathrm{meal}} = \left[\mathrm{ATTDC}\ \mathrm{X}\ \mathrm{of}\ \mathrm{insect}\ \mathrm{larvae}\ \mathrm{meal}\ \mathrm{diet}-\left(\mathrm{ATTDC}\ \mathrm{X}\ \mathrm{of}\ \mathrm{basal}\ \mathrm{diet} \times 0.75\right)\right]\ /\ 0.25. $$

where X represents DM, OM, CP, EE or GE.

The AME values of the insect larvae meals were calculated using the following formula with appropriate corrections made according to the differences in the DM content:

$$ \mathrm{AM}{\mathrm{E}}_{\mathrm{diet}}\left(\mathrm{MJ}/\mathrm{kg}\right) = \left[\left(\mathrm{feed}\ \mathrm{intake} \times \mathrm{GE}\ \mathrm{diet}\right)-\left(\mathrm{excreta}\ \mathrm{output} \times \mathrm{GE}\ \mathrm{excreta}\right)\right]\ /\ \mathrm{feed}\ \mathrm{intake} $$

$$ \mathrm{AM}{\mathrm{E}}_{\mathrm{insect}\ \mathrm{larvae}\ \mathrm{meal}}\left(\mathrm{MJ}/\mathrm{kg}\right) = \left[\mathrm{AME}\ \mathrm{of}\ \mathrm{insect}\ \mathrm{larvae}\ \mathrm{meal}\ \mathrm{diet}-\left(\mathrm{AME}\ \mathrm{basal}\ \mathrm{diet} \times 0.75\right)\right]\ /\ 0.25 $$

Correction for zero nitrogen (N) retention was made using a factor of 36.54 KJ per gram N retained in the body in order to estimate the N-corrected apparent metabolizable energy (AMEn) [27]. N-retention was calculated using the following formula:

$$ {\mathrm{N}}_{\mathrm{retention}} = \left[\left(\mathrm{feed}\ \mathrm{intake} \times \mathrm{N}\ \mathrm{diet}\right)-\left(\mathrm{excreta}\ \mathrm{output} \times \mathrm{N}\ \mathrm{excreta}\right)\right]\ /\ \mathrm{feed}\ \mathrm{intake}\ \left(\mathrm{kg}\right) $$

The AIDC of the AA of the insect larvae meals was calculated, using TiO as the indigestible marker, as follows:

$$ \mathrm{AIDC}\ \mathrm{of}\ \mathrm{AA}{\mathrm{X}}_{\mathrm{diet}} = \left(\mathrm{AAX}\ /\ \mathrm{TiO}\right)\mathrm{d}\ \hbox{--}\ \left(\mathrm{AAX}\ /\ \mathrm{TiO}\right)\mathrm{i}\ /\ \left(\mathrm{AAX}\ /\ \mathrm{TiO}\right)\mathrm{d} $$

$$ \begin{array}{l}\mathrm{AIDC}\ \mathrm{of}\ \mathrm{AA}{\mathrm{X}}_{\mathrm{insect}\ \mathrm{larvae}\ \mathrm{meal}} = \Big[\left(\mathrm{AIDC}\ \mathrm{AA}\mathrm{X}\ \mathrm{of}\ \mathrm{the}\ \mathrm{insect}\ \mathrm{larvae}\ \mathrm{meal}\ \mathrm{diet} \times \mathrm{AA}\mathrm{X}\ \mathrm{of}\ \mathrm{the}\ \mathrm{insect}\ \mathrm{larvae}\ \mathrm{meal}\ \mathrm{diet}\right)\\ {}\hbox{--}\ \left(\mathrm{AIDC}\ \mathrm{AA}\mathrm{X}\ \mathrm{of}\ \mathrm{the}\ \mathrm{basal}\ \mathrm{diet} \times \mathrm{AA}\mathrm{X}\ \mathrm{of}\ \mathrm{the}\ \mathrm{basal}\ \mathrm{diet} \times 0.75\right)\Big]\ /\ \left(\mathrm{AAX}\ \mathrm{of}\ \mathrm{the}\ \mathrm{insect}\ \mathrm{larvae}\ \mathrm{meal}\ \mathrm{diet} \times 0.25\right).\end{array} $$

where:

$$ \left(\mathrm{AA}\ /\ \mathrm{Ti}\mathrm{O}\right)\mathrm{d} = \mathrm{ratio}\ \mathrm{of}\ \mathrm{the}\ \mathrm{AA}\ \mathrm{and}\ \mathrm{T}\mathrm{i}\mathrm{O}\ \mathrm{concentrations}\ \mathrm{i}\mathrm{n}\ \mathrm{the}\ \mathrm{diet}; $$

$$ \left(\mathrm{AA}\ /\ \mathrm{AIA}\right)\mathrm{i} = \mathrm{ratio}\ \mathrm{of}\ \mathrm{the}\ \mathrm{AA}\ \mathrm{and}\ \mathrm{T}\mathrm{i}\mathrm{O}\ \mathrm{concentrations}\ \mathrm{i}\mathrm{n}\ \mathrm{the}\ \mathrm{i}\mathrm{leal}\ \mathrm{digesta}; $$

$$ \mathrm{A}\mathrm{A}\mathrm{X}:\ \mathrm{represents}\ \mathrm{each}\ \mathrm{A}\mathrm{A}\ \mathrm{evaluated}. $$

### Statistical analyses

The statistical analysis of the ATTDC, AME and AIDC of BSFp and BSFh meals was performed with SPSS 17 for Windows (SPSS, Inc., Chicago, IL, USA 2008) [28]. The experimental unit was the cage. Data were analyzed using Mann–Whitney *U* test. Before testing for group differences, non-normality of the data distribution and homogeneity of variances were assessed using the Shapiro-Wilk and Levene test, respectively. Differences were considered to be significant at *P* ≤ 0.05. A statistical trend was considered at *P* ≤ 0.10.

## Results

### Chemical and amino acid compositions of diets and insect meals

The proximate composition and GE of the two BSF meals and the three experimental diets are summarized in Table 2. The chemical composition of the two BSF meals differed mainly in terms of CP and EE contents. As expected, the BSFp meal showed a lower CP content than the BSFh meal (553 and 655 g/kg DM, respectively). On the contrary, the BSFh meal, being highly defatted, showed a lower EE content than the BSFp meal (46 and 180 g/kg DM, respectively). The GE contents of the BSFp and BSFh meals were 24.4 and 21.2 MJ/kg DM, respectively. The two defatted BSF meals revealed a relevant chitin content (50 and 69 g/kg DM for BSFp and BSFh, respectively).

**Table 2:** Analyzed chemical composition of the two BSF meals and of the three experimental diets

Items	BSFp meal	BSFh meal	Basal diet	BSFp diet	BSFh diet
Dry matter, g/kg diet	942	985	889	897	906
Organic matter, g/kg DM	901	907	814	817	824
Crude protein, g/kg DM	553	655	246	343	374
Ether extract, g/kg DM	180	46	71	103	72
Gross energy, MJ/kg DM	24.4	21.2	18.7	20.0	19.4
Chitin, g/kg DM	50	69	-	17	21

The AA compositions of the two BSF larvae meals and of the three experimental diets are presented in Table 3. As expected, the AA content was different in the two BSF meals, and BSFh showed higher content than BSFp for all AA (both indispensable and dispensable). In both meals, leucine was the most abundant indispensable AA (28.6 and 36.7 g/kg DM in BSFp and BSFh, respectively), whereas glutamic acid was the most abundant dispensable one (48.7 and 63.7 g/kg DM in BSFp and BSFh, respectively). Both meals were deficient of cysteine (0.1 and 0.2 g/kg DM in BSFp and BSFh, respectively). BSFh, having higher CP content than BSFp, was the highest source of lysine, threonine and methionine (25.2, 21.8 and 8.56 g/kg DM, respectively).

**Table 3:** Amino acid concentration (g/kg DM) of the two BSF meals and of the three experimental diets

Items	BSFp meal	BSFh meal	Basal diet	BSFp diet	BSFh diet
Indispensable amino acids					
Arginine	21.5	27.0	14.5	16.0	17.7
Histidine	12.3	16.3	5.73	7.64	8.66
Isoleucine	18.5	24.0	9.27	12.0	13.1
Leucine	28.6	36.7	17.8	20.6	22.9
Lysine	21.0	25.2	12.1	14.5	15.8
Methionine	6.46	8.56	2.43	3.52	4.46
Phenylalanine	16.6	21.8	10.5	12.0	13.4
Threonine	17.2	21.8	8.06	11.0	12.1
Valine	27.2	34.5	10.2	15.4	16.7
Dispensable amino acids					
Alanine	34.5	43.7	10.2	17.8	20.1
Aspartic acid	37.2	48.8	22.5	26.6	30.0
Cysteine	0.1	0.2	2.49	2.35	2.74
Glycine	23.5	30.3	9.03	13.6	15.3
Glutamic acid	48.7	63.7	40.1	41.4	46.2
Proline	30.6	32.7	12.6	16.1	19.0
Serine	20.3	26.8	11.8	14.6	16.5
Tyrosine	26.4	34.1	5.84	11.5	13.5

### Apparent nutrient digestibility

The ATTDC of the nutrients, as well as the AME and AMEn of the BSFp and BSFh meals are reported in Table 4. The BSFp meal resulted more digestible than the BSFh one. Significant differences were found between the two BSF meals for the ATTDC of EE and GE (*P* < 0.05), a statistical trend was observed for the ATTDC of DM and OM (*P* < 0.10), while no significant difference was found for ATTDC of CP (*P* = 0.834).

**Table 4:** Apparent digestibility coefficients of the total tract (ATTDC) of the nutrients, AME and AMEn of the two BSF meals for broilers (*n =* 5)

Items	BSFp meal	BSFh meal	SEM	*P-*value
Dry matter	0.63	0.59	0.010	0.092
Organic matter	0.69	0.64	0.012	0.057
Crude protein	0.62	0.62	0.019	0.834
Ether extract	0.98	0.93	0.951	0.008
Gross energy	0.61	0.50	0.021	0.012
AME, MJ/kg DM	16.25	11.55	0.811	0.008
AMEn, MJ/kg DM	14.87	9.87	0.860	0.008

*AME* apparent metabolizable energy, *AMEn* N-corrected apparent metabolizable energy

### Apparent metabolizable energy

The two BSF meals also differed (*P* < 0.05) for AME and AMEn (Table 4). In particular, BSFp showed mean AME and AMEn values of 16.25 and 14.87 MJ/kg DM, respectively, while for BSFh, the values of AME and AMEn were 11.55 and 9.87 MJ/kg DM, respectively.

### Apparent ileal amino acid digestibility

The determined values for the AIDC of the AA are shown in Table 5. The AIDC of the AA in the BSFp meal ranged from 0.44 to 0.92, while in the BSFh meal values ranged from 0.45 to 0.99. Overall, no significant differences were found between the two BSF meals for the AIDC of indispensable AA. The AIDC of lysine was 0.80 for both BSF meals while the AIDC of methionine were 0.83 and 0.78 for BSFp and BSFh, respectively. Among the dispensable AA, no significant differences were found except for glutamic acid (0.81 vs. 0.85), proline (0.65 vs. 0.82) and serine (0.71 vs. 0.77) that were more digestible in the BSFh meal (*P* < 0.05).

**Table 5:** Apparent ileal digestibility coefficients (AIDC) of amino acids of the two BSF meals for broilers (*n =* 5)

Items	BSFp meal	BSFh meal	SEM	*P-*value
Indispensable amino acids				
Arginine	0.79	0.80	0.015	0.841
Histidine	0.64	0.63	0.022	0.834
Isoleucine	0.83	0.87	0.018	0.293
Leucine	0.84	0.89	0.019	0.141
Lysine	0.80	0.80	0.013	0.753
Methionine	0.83	0.78	0.023	0.293
Phenylalanine	0.82	0.86	0.020	0.249
Threonine	0.73	0.77	0.022	0.675
Valine	0.90	0.91	0.015	0.833
Mean	0.80	0.81	0.016	0.917
Dispensable amino acids				
Alanine	0.92	0.99	0.026	0.249
Aspartic acid	0.82	0.80	0.012	0.234
Cysteine	0.44	0.45	0.017	0.834
Glycine	0.66	0.65	0.020	0.548
Glutamic acid	0.81	0.85	0.013	0.046
Proline	0.65	0.82	0.041	0.008
Serine	0.71	0.77	0.019	0.035
Tyrosine	0.92	0.95	0.019	0.833
Mean	0.74	0.79	0.014	0.114
Overall mean^a^	0.77	0.80	0.015	0.673

^a^Average digestibility of 17 amino acids

## Discussion

To the best of our knowledge, this study is the first one testing the ATTDC of nutrients, the AME and AMEn, and the AA AIDC of a partially defatted and a highly defatted black soldier fly larvae meal for broiler chickens.

### Chemical and amino acid compositions of diets and insect meals

The compositional data of our study showed that the two BSF meals obtained after defatting are good sources of dietary protein. Both meals showed a higher CP content than soybean meal. In particular, the BSFh meal with its 655 g/kg of DM, was close to the CP content of meat and fish meals [29, 30]. The BSFp meal had similar CP content of a defatted BSF larvae meal reported by Cullere et al. [31]. The EE content found in this experiment for BSFp was higher than those reported by Cullere et al. [31] who used defatted BSF larvae meals (156 g/kg DM) in broiler quails. These differences could be mainly due to the insect rearing substrate [6, 32] and the defatting process [33, 34], which can both influence the variability in the amounts of EE. Using three rearing substrates differing in their nutrient composition [mixture of middlings (EE: 5.9% DM), dried distillers’ grains with solubles (EE: 8.4% DM), and dried sugar beet pulp (EE: 1.1% DM)], Tschirner and Simon [35] reported that the EE content of BSF larvae reached 30.8%, 38.6% and 3.4% of DM, respectively. Fasakin et al. [14] also showed that defatting maggot meal, either oven dried or sun dried, definitely influenced its nutrient composition (EE contents varied from 7.00% to 7.40% DM, respectively). Kroeckel et al. [13] used a defatted BSF meal obtained by cutting the frozen pupae to enable the leakage of intracellular fat from the larvae using tincture press (pression at 450 bar and 60 °C for 30 min). These authors reported that the EE and CP contents for such a BSF meal were 118 and 475 g/kg DM, respectively, with high methionine and lysine contents (21.8 and 71.2 g/kg CP). Tschirner and Simon [35] showed that the best fat reduction of BSF larvae (from 30.8 to 16.6% of DM) was achieved by pressing them at 250 bar and 50 °C for 30 min. In addition, Purshke et al. [33] demonstrated that pressure, time and their interaction had the most significant effects on defatting mealworm (*Tenebrio molitor* L.) larvae. In the same context, Sheppard et al. [36] stated that the CP content of larvae meal could be increased by over 60% when protein and fat are separated. According to Castell [37], the defatting process increases the CP and decreases the lipid value of the insect meal, which may lead to total or partial destruction of AA such as cysteine, tryptophan and methionine. Our study showed that the only difference between the two meals was the fat content and that the AA concentration proportionally grew in relation to the CP content.

Based on the current knowledge, very few studies are available in the literature on the chitin content of defatted BSF larvae meals. Kroeckel et al. [13] reported a chitin amount of 96 g/kg DM in defatted BSF larvae. However, Diener et al. [38] indicated that the exoskeleton of the BSF pre-pupae contains approximately 87 g/kg DM of the polysaccharide chitin. Our results on chitin contents of both BSFp and BSFh were lower than those reported by the above mentioned authors.

Our results also confirmed that the AA of highly or partially defatted BSF meals were higher than those reported for full-fat BSF ones [12, 39]. As expected, in the defatted meal the protein was more concentrated and therefore the amount of AA was higher. In fact, the AA level in BSFp was about 20% lower than in BSFh meal. The relative percentage of each AA remained unchanged in both BSFp and BSFh meals compared with full-fat BSF meal [6, 39]. Performic acid oxidation was not performed prior to acid hydrolysis, so the values of cysteine and methionine could be undervalued.

In their study about full-fat BSF meal, De Marco et al. [12] concluded that AA level in BSF meal was lower than in other insect meals. The results obtained in the current trial showed that the defatting process managed to match, and even surpass, the AA profile of other insect meals, for example obtainable from *T. molitor* [12] and other species of insects rich in AA [5, 6, 32, 40].

AA in both defatted BSF meals used in this trial were at higher levels than in plant protein sources (pea protein, full-fat soya bean, sunflower meal and soya bean meal) [20, 30, 41, 42]. The BSFh meal showed similar levels of some AA (valine, methionine, lysine, aspartic acid, serine and alanine) than those found in meat meal (26.9–29.0; 8.1–10.2; 24.6–30.0; 41.5–45.7; 25.6–26.4; 38.8–43.5 g/kg DM, respectively) [20, 30]. In BSFh meal, the amounts of some AA (histidine, alanine, tyrosine and valine) were higher than those found for fishmeal (13.9–16.2; 40.5–41.4; 19.9–21.5; 30.4–33.0 g/kg DM, respectively), while the rest of AA remained at levels below those usually found in fishmeal [20, 30]. Therefore, the insect meal defatting process provided a new ingredient characterized by high quality AA profile. Such profile is comparable to that of other animal protein sources, namely meat and fish meal, usually used in poultry diets.

### Apparent nutrient digestibility

In the present study, significant differences were found between BSFp and BSFh meals in the ATTDC for EE and GE and a statistical trend was observed for the ATTDC of DM and OM. Nevertheless, no differences were found for ATTDC of CP. Overall, the ATTDC of the nutrients were moderate for the two defatted BSF larvae meals, except for EE. Furthermore, the ATTDC of EE in our study was comparable to that reported by De Marco et al. [12] for full-fat BSF meal. Our results are also in line with those reported by Cullere et al. [31] who found EE apparent digestibility of BSF diets close to 90% in broiler quails. Till now few researches have dealt with the use of defatted BSF meals in animal nutrition. Regarding the use of defatted insect meals in fish feed, Kroeckel et al. [13] reported that at inclusion levels higher than 33%, a defatted BSF pre-pupae meal decreased diet palatability, protein digestibility and growth performance of juvenile turbot if compared to a control diet.

Fasakin et al. [14] indicated that defatting maggot meal enabled an increase of its dietary inclusion level without affecting the growth performance of African catfish. However, a recent study on Atlantic salmon showed that highly defatted BSF meal (170 g/kg DM), dried at a conventional temperature, surprisingly reduced fish growth compared to a slightly defatted BSF meal (255 g/kg DM) dried at a low temperature [15]. The effectiveness of the defatted BSF meal seems related to a better efficient nutrient digestion, even if the metabolic mechanisms related to this effect require a more in-depth investigation. Regarding poultry, Cullere et al. [31] showed that a defatted BSF meal could be introduced in the diet for growing broiler quails at 10–15%, partially replacing conventional soybean meal and soybean oil, with no negative effects on productive performance, mortality and carcass traits. Elwert et al. [43] demonstrated that increasing levels of defatted BSF meal resulted in decreasing feed consumption and body weight of broiler chickens, but no significant effect on feed conversion ratio was observed. Recently Schiavone et al. [44] reported that the partial or total replacement of soybean oil by BSF fat in broiler diets did not affect growth performance, feed choice and carcass characteristics, while the fatty acid profile of breast meat was greatly affected by the BSF fat inclusion level. All these researchers reported interesting results about the suitability of defatted BSF meals as diet ingredients for poultry and different fish species.

Although chickens have been shown to produce chitinase in the proventriculus and hepatocytes [45], the digestibility of chitin seems to be limited [46]. The moderate ATTDC of the nutrients in the present study can be attributed to the level of chitin supplied by the BSF meals, which was reported to inhibit nutrient absorption from the intestinal tract and thereby to reduce fat and protein absorption in broiler chickens [2, 47–49]. In this context, De Marco et al. [12] speculated that the chitin contained in the exoskeleton of the BSF meal may negatively influence the apparent digestibility coefficient of the total tract of nutrients in broiler chickens. Indeed, Marono et al. [50] indicated that chitin is the main factor affecting the in vitro protein digestibility of BSF meal and showed that CP digestibility was negatively correlated to the chitin content. Moreover, it has been shown that high concentrations (up to 45%) [51] of the chitin present in the cuticular exoskeleton of insects negatively affect the feed intake and reduce protein digestibility [52].

### Apparent metabolizable energy

Regarding AME, the BSFp meal showed significantly higher levels of AME and AMEn than the BSFh meal, due to its higher content of EE. De Marco et al. [12] reported a higher level of AME and AMEn in full-fat BSF larvae meal. Elwert et al. [43] calculated the content of AMEn in three different defatted BSF meals at three different levels of fat. The data obtained showed AMEn of 12.3, 12.4 and 12.7 MJ / kg, respectively. In view of these results, the AME and AMEn values of defatted BSF are relevant, and this can result in higher economic efficiency in broiler meat production.

### Apparent ileal amino acid digestibility

Broilers were able to utilize the AA of both partially and highly defatted BSF meals with high AIDC (about 80%) and, as expected, AIDC were not affected by the defatting process, except for proline. AIDC for AA in the defatted BSF meals were similar to those reported for soybean by Valencia et al. [42], Ravindran et al. [20] and Huang et al. [53] for 21, 42 and 49 d old broiler chickens, respectively. Also, the results of AIDC were within the range of values reported by Valencia et al. [42] for protein concentrates (pea protein and soya protein concentrates). Furthermore, in the present study the AIDC of AA were higher than those reported for other animal protein sources (feather meal, meat meal and meat and bone meals) and were similar to those obtained for fish meal [20], also for the most limiting AA such as lysine, methionine and threonine. Histidine was the AA which showed the lowest AIDC for the defatted BSF meals; however, the obtained value was similar to that reported for other animal protein sources [20]. The high content of AA with good AIDC indicates that the tested defatted BSF meals could find a place as invaluable protein-rich sources in broiler feeding.

## Conclusions

The findings of this study suggest that partially and highly defatted BSF larvae meals can be suitable ingredients for broiler chickens diets. The two tested BSF meals, being rich in AA with high AIDC, could find a place as a valuable AA rich resource in the feed industry for broiler chickens. Furthermore, this study provides data on the apparent metabolizable energy of BSF meals that is helpful in the formulation of broiler diets. However moderate ATTDC of nutrients was observed, consequently digestibility issues require further studies, as the chitin content may not be the only factor able to affect nutrient digestibility. Regarding the chemical composition, BSFh resulted more appealing than BSFp due to its higher protein content. Furthermore BSFh, containing a lower EE amount, would be less prone to lipid oxidation than BSFp, which may improve the storage time of the meal. Further research efforts are necessary necessary to deeply investigate the impact of defatted larvae meal on broiler chickens growth performance, meat quality traits and consumer acceptance.

## Abbreviations

AA: Amino acid
AIDC: Apparent ileal digestibility coefficient
AME: Apparent metabolizable energy
AMEn: N-corrected apparent metabolizable energy
ATTDC: Apparent total tract digestibility coefficients
BSFh: Highly defatted black soldier fly larvae meal
BSFp: Partially defatted black soldier fly larvae meal
CP: Crude protein
d: Day
DM: Dry matter
EE: Ether extract
GE: Gross energy
OM: Organic matter
TiO: Titanium oxide
UA: Uric acid
